# The QOLA study: psychometric validation of the Quality of Life of Parents of Children with Achondroplasia (QOLA) questionnaire - a multinational study in Germany, Italy, and Portugal

**DOI:** 10.1186/s41687-026-01207-w

**Published:** 2026-09-25

**Authors:** Adekunle Adedeji, Stefanie Witt, Lea Maric, Maria Salomao, Florian Innig, Inês Alves, Chiara Provasi, Marco Sessa, Klaus Mohnike, Julia Quitmann

**Affiliations:** 1 Faculty of Social Work and Early Childhood Education, HAW Hamburg, Hamburg, Germany; 2 HAW Hamburg, Competence Center Gesundheit (CCG), Hamburg, Germany; 3 Federal Association for People of Short Stature and their Families (Bundesverband Kleinwüchsige Menschen und ihre Familien e.V.), Bremen, Germany; 4 National Patient Organisation for Skeletal Dysplasias - ANDO, Évora, Portugal; 5 School of Health and Human Development, University of Évora, Evora, Portugal; 6 Italian Association on Achondroplasia (Associazione per l Informazione e lo studio dell acondroplasia) AISAC, Milan, Italy; 7 Children s Hospital, Otto-von-Guericke-Universität Magdeburg, Magdeburg, Germany; 8 Federal Association for People of Short Stature and their Families (Bundesverband Kleinwüchsige Menschen und ihre Familien e.V.), Bremen, Germany

**Keywords:** Achondroplasia, Caregiver quality of life, Psychometric validation, Patient-reported outcomes, Cross-national study

## Abstract

**Background:**

Parents of children with achondroplasia experience multidimensional impacts on quality of life (QoL), yet validated condition-specific instruments for assessing these outcomes across countries are scarce. This study evaluated the psychometric properties of the Quality of Life of Parents of Children with Achondroplasia (QOLA) questionnaire in three European settings.

**Methods:**

A multinational, multicentre observational psychometric validation study was conducted in Germany, Italy, and Portugal. Parents/primary caregivers (*N* = 149) completed the 43-item QOLA (eight conceptual domains) and the EUROHIS-QOL-8 at baseline (T1); a subsample completed follow-up assessments (T2; *n* = 77) for test–retest analyses. Data quality (missingness, floor/ceiling effects), internal consistency (Cronbach’s α; composite reliability), temporal stability (Pearson correlations; intraclass correlation coefficients), and convergent validity (correlations with EUROHIS-QOL-8) were examined. Exploratory factor analysis informed item reduction and domain aggregation, followed by confirmatory factor analysis of the reduced model.

**Results:**

Item-level missingness was minimal, and no substantial floor/ceiling effects were observed. Eight-domain scores showed acceptable to excellent internal consistency (α = 0.706–0.905) and moderate-to-good temporal stability (single-measure ICCs = 0.64–0.75). Convergent validity was supported by negative associations between QOLA domains and generic QoL (*r* = .15–0.69). Exploratory and confirmatory analyses supported a parsimonious three-factor, 20-item model, comprising Healthcare System Trust, Caregiving Burden and Daily Impact, and Worries About the Future and Social Relationships, with acceptable fit (CFI = 0.935, TLI = 0.925, RMSEA = 0.066), strong reliability (α = 0.852–0.918; CR = 0.864–0.914), and good test–retest stability (*r* = .670–0.782; average-measure ICCs = 0.801–0.877).

**Conclusions:**

The QOLA demonstrates robust reliability and validity across three countries and offers both an eight-domain profile and a three-factor summary for assessing parental QoL in achondroplasia.

**Supplementary Information:**

The online version contains supplementary material available at https://doi.org/10.1186/s41687-026-01207-w.

## Background

Parents of children with chronic and rare conditions assume sustained caregiving responsibilities that extend well beyond routine parenting and have profound implications for parental well-being and family functioning. Caregiving burden is a central determinant of parental quality of life and is increasingly conceptualised as a multidimensional construct encompassing physical strain, emotional exhaustion, time pressure, and disruptions to occupational and social roles. Cousino and Hazen [6], Raina [22]. In clinical contexts, these burdens are often cumulative and persistent, reflecting long-term care trajectories rather than discrete episodes of illness management.

Achondroplasia is the most common form of skeletal dysplasia and the leading genetic cause of disproportionate short stature, resulting from gain-of-function mutations in the fibroblast growth factor receptor 3 (FGFR3) gene [11]. With an estimated prevalence of 1 in 10,000–30,000 live births, achondroplasia is characterised by rhizomelic limb shortening, macrocephaly, midface hypoplasia, and a range of orthopaedic and neurological complications, including spinal stenosis, foramen magnum compression, obstructive sleep apnoea, and recurrent otitis media [11, 19]. These features necessitate lifelong, multidisciplinary medical follow-up beginning in infancy.

The clinical and functional implications of achondroplasia extend beyond affected individuals to their families. Parents are typically responsible for coordinating complex care pathways, managing recurrent medical appointments and interventions, supporting mobility and activities of daily living, and adapting physical and social environments to the child’s needs [26]. Empirical studies in rare paediatric conditions indicate that such demands often interfere with employment, social participation, and family routines, contributing to sustained caregiving burden and reduced parental quality of life over time [20, 22].

Beyond day-to-day caregiving tasks, parents frequently report persistent future- and social-related worries. These include concerns about the child’s long-term independence, educational and vocational opportunities, social inclusion, and potential exposure to stigma or discrimination. Theoretical models of family adaptation to chronic illness highlight anticipatory stress as a distinct dimension of caregiving burden, independent of current disease severity [23]. Empirical evidence supports the relevance of such future-oriented concerns as contributors to parental psychological strain in rare and chronic childhood conditions [2, 15].

Interactions with healthcare systems further shape parental experiences and well-being. Parents of children with achondroplasia often function as long-term care coordinators within fragmented systems involving multiple specialists and services. Parental well-being has been linked to perceptions of professional expertise, continuity of care, accessibility of specialised services, and experiences of being recognised as partners in decision-making [20, 27]. Difficulties in navigating care structures or repeated advocacy demands may undermine trust in healthcare systems and exacerbate overall caregiving burden.

Despite recognition of these multidimensional challenges, research on parental outcomes in achondroplasia remains limited, and existing studies rely predominantly on generic caregiver burden or quality-of-life instruments. Such measures do not specifically assess condition-specific dimensions, including daily-life constraints associated with physical and functional differences, anticipatory psychosocial strain related to the child’s future functioning, and trust in the healthcare system, raising concerns about construct underrepresentation and limited clinical interpretability [28]. Thus, while generic measures provide valuable information for cross-condition comparisons, condition-specific measures can complement them by capturing the particular impact of a condition on HRQoL outcomes (Adedeji et al., 2024).

This distinction motivated the development of the Quality of Life of Parents of Children with Achondroplasia (QOLA) questionnaire as a condition-specific parent-reported outcome measure. The present study evaluates the psychometric properties of the QOLA in a multinational European sample to establish its reliability and validity for use in research, clinical assessment, and service evaluation.

## Methods

### Study design

A multinational, multicentre observational psychometric validation study was conducted to evaluate the Quality of Life of Parents of Children with Achondroplasia (QOLA) questionnaire in Germany, Italy, and Portugal. The design comprised a cross-sectional field test and a test–retest component, in accordance with established guidelines for validating patient- and proxy-reported outcome measures [17, 21]. At baseline (T1), participants completed the full QOLA questionnaire to assess internal consistency, structural validity, and construct validity. A subsample completed the questionnaire again after a predefined interval (T2) to assess test–retest reliability. No intervention occurred between measurement occasions. Study procedures were harmonised across countries.

### Participants

Participants were parents or primary caregivers of children with a clinically confirmed diagnosis of achondroplasia. We recruited participants in Germany, Italy, and Portugal, aiming to enrol approximately 50 participants per country, in line with recommended sample sizes for psychometric validation studies in rare disease populations. In Germany, participants were recruited through the Bundesverband Kleinwüchsige Menschen und ihre Familien e.V. (BKMF). In Italy, AISAC APS (Associazione Italiana Sostegno Achondroplasia) facilitated recruitment, and in Portugal, ANDO Portugal (Associação Nacional de Displasias Ósseas). These organisations disseminated study information via established communication channels, including mailing lists, newsletters, and online platforms.

Eligible participants were adults (≥ 18 years) who identified as parents or primary caregivers of a child with achondroplasia and were able to complete the questionnaire in the respective national language (German, Italian, or Portuguese). No exclusion criteria were applied about the child’s age, disease severity, or treatment status, to ensure broad representation of caregiving experiences. Participation was voluntary, and all respondents provided written informed consent prior to participation. As compensation for their time and contributions, participants received a €30 gift card upon completion of the questionnaire. Ethical approval was obtained in accordance with national and institutional requirements in each participating country.

### Measures

#### Quality of Life of Parents of Children with Achondroplasia (QOLA)

Parental quality of life was assessed using the Quality of Life of Parents of Children with Achondroplasia (QOLA) questionnaire [1], a condition-specific parent-reported outcome measure developed to capture the multidimensional impact of raising a child with achondroplasia. The QOLA was developed through a multi-stage process that included qualitative interviews with parents, expert consultation, and reference to established conceptual frameworks of caregiver burden, family functioning, and quality of life in rare paediatric conditions. Items address experiences related to the child’s condition and its consequences for parental physical, emotional, social, and functional well-being [1]. The early-stage version of QOLA was developed in German, and consequently, all language versions underwent forward translation by bilingual researchers with expertise in health measurement and the subject matter, followed by two successive international card-sorting exercises to assess conceptual coherence, cross-cultural interpretability, and content validity, with iterative refinement based on participant feedback [1].

The tested version of the questionnaire comprises 43 items organised into 8 conceptual domains that reflect theoretically distinct dimensions of parental quality of life. These domains capture Healthcare System Trust (4 items), Care System Strain (5 items), Physical Strain of caregiving (5 items), Mental Health and Emotional Burden (6 items), Social Integration and Participation (5 items), Family Dynamics and Support (7 items), Coping (5 items), and Worries About the Future and Social Relationships (6 items). After appropriate reverse coding, higher scores indicate a more negative experience within the respective dimension. Higher scores across dimensions therefore indicate a more adverse parental experience, which is interpreted as indicative of poorer parental quality of life, whereas lower scores indicate a less negative parental experience and better parental quality of life.

To enhance data quality and reduce acquiescence bias, the questionnaire intentionally includes both positively and negatively worded items. This mixed-item directionality was implemented to encourage attentive responding and minimise response sets. All negatively phrased items were reverse-coded prior to analysis to ensure consistent interpretation, with higher scores reflecting greater burden/impairment.

#### Generic quality of life measure (EUROHIS-QOL 8)

To assess convergent validity, participants were also asked to complete the EUROHIS-QOL-8 questionnaire at baseline (T1) and follow-up (T2). Comparison between the measures was intended to examine whether QOLA scores were meaningfully related to the broader construct of quality of life, while retaining sensitivity to condition-specific experiences not captured by generic measures.

The EUROHIS-QOL 8 is a widely used generic quality-of-life instrument derived from the WHOQOL framework and comprises 8 items covering overall quality of life, general health, energy, daily activities, self-esteem, personal relationships, financial resources, and living conditions. Each item is rated on a five-point Likert scale, with higher scores indicating better overall quality of life. A total score can be computed by summing or averaging item responses, with higher values reflecting a higher perceived generic quality of life. The EUROHIS-QOL 8 has demonstrated good psychometric properties across diverse populations and cultural contexts and was included to examine the relationship between condition-specific and generic quality-of-life constructs.

#### Sociodemographic and socioeconomic variables

Data on participants’ demographic and socioeconomic characteristics were collected during the field test to describe and characterise the study sample. Information was obtained via self-report and included the respondent’s identity, age, gender, marital status, highest level of education attained by the respondent and their spouse or partner, current employment status, average monthly household income, and the number of children living in the household. These variables were collected to provide a comprehensive description of the sample and to contextualize the psychometric findings.

### Data analysis

Data analyses were conducted in a stepwise manner to describe the study sample, examine cross-national comparability, and evaluate the psychometric properties of the Quality of Life of Parents of Children with Achondroplasia (QOLA) questionnaire. Analyses followed established recommendations for validating patient- and proxy-reported outcome measures, particularly those of the COSMIN initiative [17, 21]. Statistical analyses were performed using SPSS (version 31) and AMOS for structural equation modelling.

Descriptive statistics (means, standard deviations, and response distributions) were calculated for all QOLA items and domain scores at baseline (T1) to assess data completeness, variability, and floor or ceiling effects [7, 29]. Cross-national comparability of QOLA domain scores across Germany, Italy, and Portugal was examined using analysis of variance or non-parametric alternatives when distributional assumptions were violated. These analyses were intended to screen for significant country effects and to support data pooling, rather than to test measurement invariance formally [3, 30].

Convergent validity was assessed by calculating Pearson correlations between QOLA domain scores and the EUROHIS-QOL-8 total score at baseline (T1). Although the QOLA comprises distinct, condition-specific domains, these represent theoretically related aspects of the broader construct of parental quality of life assessed by the EUROHIS-QOL, supporting its use for convergent validity. Where follow-up data were available, correlations were additionally examined at T2 to evaluate the temporal stability of these associations [4, 28].

Internal consistency of the original eight-domain structure was evaluated using Cronbach’s alpha (≥ 0.70 considered acceptable) and corrected item–total correlations [18]. Inter-domain correlations were calculated to examine relationships among domains. Temporal stability (test–retest reliability) was evaluated in the retest subsample using intraclass correlation coefficients (ICCs) from two-way mixed-effects models with absolute agreement, supplemented by Pearson correlations between T1 and T2 scores for interpretability [14, 25].

Exploratory factor analysis (EFA) was conducted at the item level to examine the questionnaire’s latent structure and evaluate whether a more parsimonious factor solution could be identified. Factor extraction was guided by eigenvalues > 1, inspection of the scree plot, and conceptual interpretability, using oblique rotation to allow correlated factors [5, 8]. Based on EFA results, a reduced three-factor model was derived and subsequently evaluated using confirmatory factor analysis (CFA) in AMOS. Model fit was assessed using the Comparative Fit Index (CFI), Tucker–Lewis Index (TLI), and Root Mean Square Error of Approximation (RMSEA), with established cut-off values [12, 13].

For the final factor structure, internal consistency was evaluated using Cronbach’s alpha and composite reliability (CR). Convergent validity was examined using average variance extracted (AVE), and discriminant validity was assessed using maximum shared variance (MSV) and average shared variance (ASV) [9, 10]. Test–retest reliability of factor scores was assessed using ICCs, and Pearson correlations between T1 and T2 scores; ICC values ≥ 0.70 were interpreted as indicating good temporal stability.

## Results

### Participant characteristics

A total of 149 parents or primary caregivers of children with achondroplasia completed the baseline assessment (T1). Participants were recruited in Germany (*n* = 55, 37.2%), Italy (*n* = 43, 29.1%), and Portugal (*n* = 50, 33.8%); one participant with missing country information was excluded from country-specific analyses. Baseline demographic and socioeconomic characteristics of the full sample and the retest subsample are presented in Table 1.

**Table 1:** Presents detailed baseline demographic and socioeconomic characteristics for the full sample and the retest subsample

Characteristic	Baseline sample (T1), *N* = 149	Retest subsample (T2), *N* = 77
Country		
Germany	55 (37.2%)	14 (18.2%)
Italy	43 (29.1%)	25 (32.5%)
Portugal	50 (33.8%)	38 (49.4%)
Respondent		
Mother	111 (74.5%)	57 (74.0%)
Father	38 (25.5%)	20 (26.0%)
Gender		
Female	110 (73.8%)	57 (74.0%)
Male	39 (26.2%)	20 (26.0%)
Age (years)		
Range	27–67 (M = 44.12 SD = 7.13)	28–58 (M = 44.72 SD = 6.65)
Marital status		
Married	105 (70.5%)	53 (68.8%)
In a relationship, not married	31 (20.8%)	19 (24.7%)
Single	6 (4.0%)	2 (2.6%)
Divorced	7 (4.7%)	3 (3.9%)
Education of spouse/partner		
Secondary education or lower	39 (26.7%)	26 (34.7%)
Post-secondary / vocational	30 (20.5%)	13 (17.3%)
Bachelor’s degree	37 (25.3%)	17 (22.7%)
Master’s degree or higher	40 (27.4%)	19 (25.3%)
Household income (monthly)		
< €1,500	18 (12.1%)	12 (15.6%)
€1,500–3,499	56 (37.6%)	33 (42.9%)
€3,500–5,000	30 (20.1%)	15 (19.5%)
> €5,000	37 (24.8%)	14 (18.2%)
Not specified	8 (5.4%)	3 (3.9%)

Most questionnaires were completed by mothers (74.5%), and the majority of participants identified as female (73.8%). Participants’ ages ranged from 27 to 67 years (M = 44.1, SD = 7.1). Most participants were married (70.5%) or in a relationship (20.8%). Educational attainment of spouses or partners was generally high, with approximately half holding a bachelor’s degree or higher. Household income varied widely, with 24.8% reporting a monthly income above €5,000 and 12.1% reporting a monthly income below €1,500

Seventy-seven participants (51.7%) completed the follow-up assessment (T2) and were included in test–retest analyses. The retest subsample included participants from Germany (18.2%), Italy (32.5%), and Portugal (49.4%). The demographic and socioeconomic profile of the retest subsample was comparable to the baseline sample, with similar distributions of gender, age, marital status, education, and income, indicating no evidence of substantial selective attrition between T1 and T2.

### Descriptive statistics and data quality

At baseline (T1), QOLA domain scores spanned a broad range of the available response scale, indicating substantial variability across domains (Table 2). Mean domain scores ranged from M = 1.94 (SD = 0.74) for Healthcare System Trust to M = 3.72 (SD = 0.83) for Worries about the Future and Social Relationships. Lower mean scores were observed for domains reflecting healthcare system trust and coping (e.g., Healthcare System Trust: M = 1.94, SD = 0.74; Coping: M = 2.04, SD = 0.61), indicating comparatively lower perceived burden in these areas. Higher mean scores were observed for domains capturing care-related strain and psychosocial concerns (e.g., Care System Strain: M = 3.04, SD = 0.79; Mental Health and Emotional Burden: M = 3.11, SD = 0.73), reflecting greater reported burden in these domains. Standard deviations were moderate across all domains (SD range = 0.61–0.83), indicating adequate variability without evidence of excessive dispersion.

**Table 2:** Descriptive statistics of QOLA domain scores, three-factor model, and EUROHIS-QOL-8 at baseline (T1) and retest (T2)

Measure	Items (*n*)	T1 Mean (SD)	T1 Min–Max	T2 Mean (SD)	T2 Min–Max
**QOLA domains**					
Healthcare system trust	4	1.94 (0.74)	1.00–4.50	1.96 (0.73)	1.00–4.00
Care system strain	5	3.04 (0.79)	1.00–5.00	2.97 (0.77)	1.00–5.00
Physical strain of caregiving	5	2.79 (0.76)	1.00–4.80	2.72 (0.73)	1.20–4.80
Mental health and emotional burden	6	3.11 (0.73)	1.33–4.83	3.04 (0.68)	1.50–4.67
Social integration and participation	5	2.34 (0.68)	1.00–4.40	2.35 (0.63)	1.00–4.20
Family dynamics and support	7	2.37 (0.81)	1.00–4.14	2.36 (0.77)	1.00–3.86
Coping	5	2.04 (0.61)	1.00–4.20	2.03 (0.61)	1.00–4.20
Worries about the future and social relationships	6	3.72 (0.83)	1.00–5.00	3.85 (0.78)	1.50–5.00
**QOLA three-factor model**					
F1 Healthcare system trust	4	1.94 (0.74)	1.00–4.50	1.96 (0.73)	1.00–4.00
F2 Caregiving burden and daily impact	11	2.54 (0.82)	1.00–4.45	2.52 (0.82)	1.00–4.18
F3 Worries about the future and social relationships	6	3.67 (0.84)	1.00–5.00	3.76 (0.79)	1.67–5.00
**Generic quality of life**					
EUROHIS-QOL-8 total score	8	3.65 (0.62)	1.88–4.88	3.58 (0.54)	1.88–4.63

At retest (T2), domain score distributions were highly comparable to baseline, with minimal changes in mean and standard deviation across domains, supporting the stability of score distributions over time. Observed score ranges at both measurement occasions covered a substantial portion of the scale for all domains

For the generic quality-of-life measure, the EUROHIS-QOL-8 total score indicated a moderate-to-high perceived quality of life at baseline (M = 3.65, SD = 0.62) and remained similar at retest (M = 3.58, SD = 0.54). Item-level responses demonstrated adequate spread across all five response categories. Mean item scores ranged from M = 3.40 (SD = 0.85) for energy for everyday life to M = 4.03 (SD = 0.66) for the global quality-of-life item (Supplementary Table S1).

Item-level missingness was minimal across both QOLA domains and the EUROHIS-QOL-8 items, with missingness rates generally below 1%. The only exception was the global quality-of-life item of the EUROHIS-QOL-8, which had a higher non-response rate (19.5%) due to its optional placement in the questionnaire.

Assessment of floor and ceiling effects revealed no substantial clustering at the lowest or highest possible scores. For all QOLA domain scores and the EUROHIS-QOL-8 total score, fewer than 15% of respondents achieved minimum or maximum values at either time point, meeting established criteria for acceptable measurement range and sensitivity. Distributional inspections suggested approximate normality for most domain scores. Mild skewness was observed in selected caregiving-related domains. However, it was not considered problematic given the sample size and the use of non-parametric and robust statistical methods in subsequent analyses.

### Cross-national comparability and country-specific convergent validity

Cross-national comparability of QOLA domain scores was examined at baseline (T1) and retest (T2) using Kruskal–Wallis tests due to mild deviations from normality. At T1, no significant country effects were observed for most QOLA domains, including caregiving strain, physical strain of caregiving, mental health and emotional burden, social integration and participation, and care organisation (all *p* > .05). Domain score distributions showed substantial overlap across countries, with comparable ordering of domain means. Significant country differences were confined to family dynamics and support (H(2) = 10.57, *p* = .005), worries about the future and social relationships (H(2) = 16.11, *p* < .001), and the EUROHIS-QOL-8 total score (H(2) = 11.35, *p* = .003). At T2, a highly similar pattern was observed. Most QOLA domains again showed no significant country effects (all *p* > .05). Significant differences remained limited to family dynamics and support (H(2) = 9.84, *p* = .007), worries about the future and social relationships (H(2) = 14.62, *p* < .001), and the EUROHIS-QOL-8 total score (H(2) = 10.91, *p* = .004), indicating temporal stability of cross-national comparability.

Country-specific convergent validity was examined using Pearson correlations between QOLA domain scores and the EUROHIS-QOL-8 total score for Germany, Italy, and Portugal at T1 and T2 (Supplementary Table S2). In the pooled sample, all QOLA domains were significantly negatively correlated with generic quality of life at both time points (|r| = 0.15–0.69, all *p* ≤ .031). The strongest associations were observed for physical strain of caregiving, mental health and emotional burden, and caregiving strain.

At T1, country-specific analyses showed consistent directions of association despite variability in statistical significance. In Germany, significant correlations were observed for caregiving strain, physical strain of caregiving, mental health and emotional burden, social integration and participation, care organisation, and family dynamics and support (*r* = − .32 to − 0.68, all *p* ≤ .017). In Italy, significant associations were found for caregiving strain, physical strain of caregiving, mental health and emotional burden, social integration and participation, and care organisation (*r* = − .34 to − 0.53, all *p* ≤ .027). In Portugal, significant correlations were observed for healthcare system trust, caregiving strain, physical strain of caregiving, mental health and emotional burden, social integration and participation, and family dynamics and support (*r* = − .40 to − 0.67, all *p* ≤ .004). At T2, the overall pattern was replicated across countries. Physical strain of caregiving and mental health and emotional burden remained significantly associated with generic quality of life in all three countries (Germany: *r* = − .63 and − 0.59; Italy: *r* = − .72 and − 0.63; Portugal: *r* = − .76 and − 0.44; all *p* ≤ .026). Caregiving strain and care organisation also showed significant negative associations, whereas associations involving healthcare system trust and worries about the future and social relationships were less consistently significant (See Supplementary Table S2). These findings suggest no substantial country-related differences in score distributions for most domains, supporting pooled psychometric analyses.

### Internal consistency and temporal stability of the eight-domain version

Internal consistency of the eight QOLA domains was evaluated at baseline (T1; pooled sample, *N* = 149) using Cronbach’s alpha and corrected item–total correlations (Table 3). Cronbach’s alpha coefficients ranged from α = 0.706 for Coping to α = 0.905 for Healthcare System Trust, indicating acceptable to excellent internal consistency across domains. All domains met or exceeded the conventional threshold of α ≥ 0.70 for group-level comparisons.

**Table 3:** Internal consistency and Test–Retest Reliability of the QOLA eight-domain version at baseline (T1)

Domain (T1)	Items	α	Corrected item–total *r* range	ICC (single)	95% CI	ICC (average)	Interpretation
Healthcare System Trust	4	0.905	0.723–0.856	0.68	0.53–0.78	0.81	Good
Care System Strain	5	0.779	0.431–0.640	0.64	0.49–0.76	0.78	Moderate–Good
Physical Strain of Caregiving	5	0.756	0.476–0.563	0.75	0.64–0.84	0.86	Good
Mental Health and Emotional Burden	6	0.798	0.510–0.693	0.70	0.56–0.80	0.82	Good
Social Integration and Participation	5	0.724	0.388–0.615	0.70	0.57–0.80	0.82	Good
Coping	5	0.706	0.281–0.603	0.70	0.56–0.80	0.82	Good
Family Dynamics and Support	7	0.840	0.198–0.709	0.75	0.63–0.83	0.85	Good
Worries about the Future and Social Relationships	6	0.841	0.435–0.732	0.68	0.54–0.79	0.81	Good

**Note. α** Cronbach’s alpha values ≥ 0.70 indicate acceptable internal consistency. Item–total correlations refer to corrected correlations between each item and its corresponding domain total score. ICCs are based on two-way mixed-effects models with absolute agreement (ICC[A,1]). Interpretation follows conventional thresholds: <0.50 = poor, 0.50–0.75 = moderate, > 0.75 = good reliability

Corrected item–total correlations demonstrated adequate item discrimination across domains, with values ranging from *r* = .198 to 0.856. Item–total correlation ranges were highest for Healthcare System Trust (0.723–0.856) and Worries about the Future and Social Relationships (0.435–0.732), and lowest for Family Dynamics and Support (0.198–0.709), reflecting greater heterogeneity within this domain. Overall, the pattern of item–total correlations support the internal coherence of the eight-domain conceptualisation.

Temporal stability was examined in the retest subsample of participants who completed both baseline (T1) and follow-up (T2) assessments (*n* = 77). ICCs are based on two-way mixed-effects models with absolute agreement (ICC[A,1]) (Table 3). Single-measure ICCs ranged from 0.64 to 0.75, indicating moderate to good stability over the retest interval. The highest stability was observed for Physical Strain of Caregiving and Family Dynamics and Support (both ICC = 0.75), followed by Mental Health and Emotional Burden, Social Integration and Participation, and Coping (ICCs ≈ 0.69–0.70). Slightly lower but still acceptable stability was found for Care System Strain (ICC = 0.64) and Worries about the Future and Social Relationships (ICC = 0.68). These domains may be more sensitive to short-term contextual or situational changes.

Average-measure ICCs ranged from 0.78 to 0.86, indicating good reliability across the eight domains. All ICC estimates were statistically significant (*p* < .001), and confidence intervals consistently excluded values indicative of poor stability. Taken together, these findings support both the internal consistency and temporal reliability of the original eight-domain version of the QOLA questionnaire.

### Exploratory factor analysis and item reduction

An exploratory factor analysis (EFA) was conducted to examine the latent structure underlying the QOLA item pool and to inform domain aggregation and item reduction. Given the conceptual overlap observed across several of the original eight domains and the absence of an a priori higher-order structure, EFA was considered appropriate to evaluate whether a more parsimonious factor solution could be empirically supported.

Sampling adequacy was excellent (Kaiser–Meyer–Olkin measure = 0.857), and Bartlett’s test of sphericity was statistically significant, χ² (595) = 3054.78, *p* < .001, indicating that the correlation matrix was suitable for factor analysis. Factors were extracted using principal axis factoring with oblique (Promax) rotation to allow for correlated latent dimensions.

Initial eigenvalues suggested a seven-factor solution (eigenvalues > 1); however, inspection of the scree plot and evaluation of the rotated factor pattern indicated that a smaller number of substantively interpretable factors accounted for the majority of shared variance. The first three factors showed clearly elevated eigenvalues (10.54, 4.28, and 2.53). Together, they explained 49.6% of the total variance prior to rotation, whereas subsequent factors contributed comparatively little additional explanatory value and were less interpretable. Based on eigenvalue distribution, scree plot inspection, and conceptual coherence, a three-factor solution was retained for further analysis.

The EFA was also used to guide item reduction. Items that did not show a clear primary loading on any factor or that exhibited substantial cross-loadings across multiple factors in the rotated pattern matrix were excluded. This procedure aimed to enhance structural clarity and interpretability while retaining items with strong empirical loadings and conceptual relevance. As a result, the final three-factor solution comprised a reduced subset of items relative to the original eight-domain configuration.

The retained factors reflected three conceptually distinct yet correlated dimensions of parental quality of life. Factor 1 captured items reflecting trust and confidence in the healthcare system and in the provision of professional care. Factor 2 comprised items related to caregiving strain, disruptions to daily and professional life, and reduced social participation. Factor 3 encompassed items reflecting worries about the child’s future, social relationships, and emotional concerns.

Based on the empirical clustering of items and their conceptual content, items from multiple original domains were aggregated into these three higher-order factors. This aggregation represents a shift from a fine-grained domain structure toward a more parsimonious representation of caregiver-reported quality-of-life impacts, while preserving coverage of healthcare system trust, caregiving burden and daily impact, and future-oriented psychosocial concerns.

### Confirmatory factor analysis of the three-factor model

**Fig. 1:**
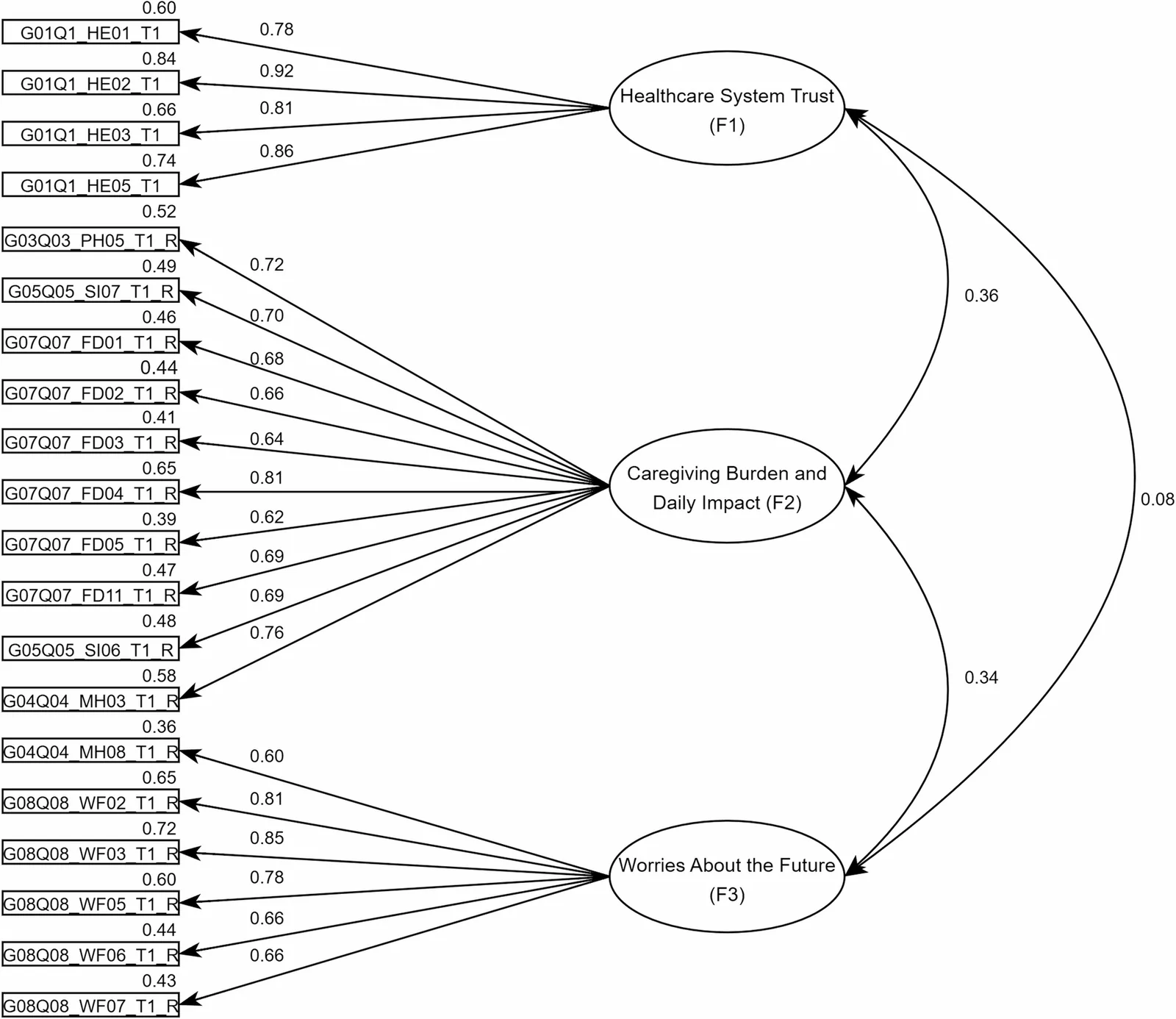
Final three-factor CFA model. Factor correlations: F1–F2 *r* = .378; F2–F3 *r* = .341; F1–F3 *r* = − .084 (ns; *p* = .359). Model fit: χ² (164) = 268.404; χ²/df = 1.637; CFI = 0.935; TLI = 0.925; RMSEA = 0.066 (90% CI 0.051–0.079); PCLOSE = 0.039

A confirmatory factor analysis (CFA) was conducted in AMOS to test the three-factor structure derived from the EFA using the final 20-item set (*N* = 149). The specified recursive model comprised three correlated latent factors: F1 (Healthcare System Trust), F2 (Caregiving Burden and Daily Impact), and F3 (Worries About the Future and Social Relationships), each indicated by the retained items (Fig. 1).

The model demonstrated acceptable overall fit: χ²(164) = 268.404, *p* < .001, χ²/df = 1.637. Incremental fit indices were in the acceptable-to-good range (CFI = 0.935, TLI = 0.925, IFI = 0.936, NFI = 0.851, RFI = 0.828), and absolute fit was supported by RMSEA = 0.066 (90% CI [0.051, 0.079], PCLOSE = 0.039). The standardised root mean square residual (SRMR) was not available in the AMOS output.

All standardised factor loadings were positive and of moderate to high magnitude (Fig. 1), ranging from 0.775 to 0.916 for F1, 0.623 to 0.805 for F2, and 0.597 to 0.848 for F3. Inter-factor correlations were small to moderate: F1 correlated with F2 (*r* = .378), F2 correlated with F3 (*r* = .341), and the association between F1 and F3 was small and non-significant (*r* = − .084, *p* = .359).

### Reliability and validity of the three-factor version

Internal consistency reliability of the three-factor version of the QOLA was evaluated using Cronbach’s alpha at baseline (T1) and retest (T2). All three factors demonstrated good to excellent internal consistency at T1, with α = 0.905 for Healthcare System Trust (F1; 4 items), α = 0.908 for Caregiving Burden and Daily Impact (F2; 11 items), and α = 0.865 for Worries About the Future and Social Relationships (F3; 6 items). Comparable reliability was observed at T2 (α = 0.878–0.918), indicating stable internal consistency across measurement occasions. In line with COSMIN recommendations, all alpha coefficients fell within the acceptable range of 0.70–0.95, indicating adequate internal consistency and no evidence of redundancy.

To complement alpha-based estimates, composite reliability (CR) was calculated from standardised factor loadings obtained from the confirmatory factor analysis. CR values were high across all three factors (CR = 0.864–0.914; Table 4), providing further evidence of strong internal reliability at the latent construct level.

Convergent validity was examined using standardised factor loadings and average variance extracted (AVE). All CFA loadings were moderate to high (0.597–0.916), indicating that retained items were strongly associated with their intended latent constructs. AVE values exceeded or closely approached the recommended threshold of 0.50 for all factors (F1 = 0.711; F2 = 0.495; F3 = 0.506). Although AVE for F2 was marginally below 0.50, this was considered acceptable given its high composite reliability and the conceptual breadth of the caregiving burden construct.

Discriminant validity was assessed using the Fornell–Larcker criterion. For all three factors, AVE values exceeded both the maximum shared variance (MSV) and the average shared variance (ASV), supporting adequate discriminant validity (Table 4). Inter-factor correlations were small to moderate, with a moderate association between Healthcare System Trust and Caregiving Burden and Daily Impact (*r* = .378), and between Caregiving Burden and Daily Impact and Worries About the Future and Social Relationships (*r* = .341). The association between Healthcare System Trust and Worries About the Future and Social Relationships was small and non-significant (*r* = − .084), indicating clear conceptual separation between system-related trust and future-oriented psychosocial concerns.

Temporal stability of the three-factor version was evaluated using Pearson correlations and ICCs from two-way mixed-effects models with absolute agreement in the retest subsample (*n* = 77). Pearson correlations between T1 and T2 factor scores indicated substantial stability over time (*r* = .670–0.782, all *p* < .001). ICC analyses further supported good temporal reliability, with single-measure ICCs ranging from 0.668 to 0.781 and average-measure ICCs ranging from 0.801 to 0.877 (Table 4). According to COSMIN criteria, these values indicate sufficient to good reliability, suggesting that the three-factor QOLA scores are stable over time while remaining sensitive to meaningful variation in parental experiences.

**Table 4:** Internal consistency and temporal stability of the QOLA three-factor model

Factor	Domain label	Items	α (T1)	α (T2)	CR	AVE	MSV	ASV	Pearson *r* (T1–T2)	ICC (single)	ICC (average)	95% CI (average)
F1	Healthcare System Trust	4	0.905	0.878	0.907	0.711	0.143	0.075	0.682***	0.675	0.806	[0.695, 0.877]
F2	Caregiving Burden and Daily Impact	11	0.908	0.918	0.914	0.495	0.143	0.130	0.782***	0.781	0.877	[0.807, 0.922]
F3	Worries About the Future and Social Relationships	6	0.865	0.852	0.864	0.506	0.116	0.062	0.670***	0.668	0.801	[0.687, 0.874]

Note: Cronbach’s α was calculated at baseline (T1) and retest (T2). Composite reliability (CR) was derived from standardised factor loadings of the confirmatory factor analysis. Average variance extracted (AVE) reflects convergent validity; maximum shared variance (MSV) and average shared variance (ASV) were used to assess discriminant validity following the Fornell–Larcker criterion. Test–retest reliability was evaluated using Pearson correlations and intraclass correlation coefficients (ICCs) based on two-way mixed-effects models with absolute agreement (ICC[A,1] for single measures; ICC[A, k] for average measures). Higher QOLA scores indicate greater burden or impairment. ***p < .001

## Discussion

This study evaluated the psychometric properties of the Quality of Life of Parents of Children with Achondroplasia (QOLA) questionnaire in accordance with COSMIN recommendations for the development and validation of patient- and proxy-reported outcome measures [17, 21]. The findings support the QOLA as a condition-specific instrument with adequate content validity, structural validity, internal consistency, and reliability for assessing parental quality of life in the context of achondroplasia. Taken together, the results substantiate a multidimensional conceptualisation of caregiving burden that integrates daily-life constraints, future- and social-related worries, and trust in the healthcare system within a coherent measurement framework. Importantly, the results substantiate a multidimensional conceptualisation of caregiving burden that integrates daily-life constraints, future- and social-related worries, and trust in the healthcare system within a coherent measurement framework.

From a content validity perspective, the QOLA addresses a key limitation of existing caregiver burden and quality-of-life instruments, which often underrepresent dimensions that are highly salient in rare paediatric conditions. COSMIN guidelines emphasise that content validity, defined as the degree to which an instrument adequately reflects the construct of interest, is the most critical measurement property [17]. The inclusion of items capturing daily caregiving demands, anticipatory psychosocial strain, and healthcare system experiences reflects both theoretical models of family adaptation to chronic illness and empirical evidence on parental experiences in rare disease contexts [20, 23]. By explicitly operationalising these domains, the QOLA reduces the risk of construct underrepresentation that has been noted for generic caregiver measures [28].

Structural validity analyses further support the conceptual framework underlying the QOLA. The identification of a coherent higher-order factor structure that aggregates related domains while preserving conceptual distinctions is consistent with COSMIN standards for evaluating dimensionality [21]. The emergence of factors reflecting caregiving burden and daily impact, future-oriented psychosocial worries, and trust in the healthcare system aligns with theoretical expectations and suggests that these dimensions constitute related yet non-redundant components of parental quality of life. This finding reinforces the argument that caregiving burden should not be treated as a unidimensional construct, particularly in conditions characterised by long-term uncertainty and complex care trajectories.

Importantly, the findings support the utility of both the original eight-domain configuration and the more parsimonious higher-order factor structure of the QOLA. The eight-domain version allows for fine-grained assessment of distinct aspects of parental experience, including specific caregiving demands, family dynamics, coping resources, and system-related challenges. This level of differentiation may be particularly valuable for clinicians conducting individual needs assessments or identifying specific areas requiring supportas well as for exploratory research aimed at identifying specific areas of burden or support. In contrast, the higher-order factor structure provides a more concise representation of parental quality of life, which may be better suited for use researchers conducting large-scale studies, intervention trials, or outcome monitoring, where parsimony and statistical efficiency are prioritised. The availability of both scoring approaches enhances the flexibility and interpretability of the QOLA and is consistent with COSMIN guidance, which recognises the value of multidimensional instruments that can be meaningfully aggregated at different levels depending on the intended application.

Internal consistency and reliability estimates indicate that the QOLA domains and factors demonstrate sufficient homogeneity without evidence of excessive item redundancy, meeting COSMIN criteria for reliability [7, 18]. The observed temporal stability further suggests that the instrument captures relatively stable aspects of parental quality of life while remaining sensitive to dimensions that may fluctuate in response to contextual or situational factors. This balance between stability and variability is considered desirable for quality-of-life measures intended for both observational and evaluative research [29].

Evidence for construct validity was supported by theoretically coherent associations with a generic quality-of-life measure. In line with COSMIN recommendations, moderate correlations were interpreted as indicative of related but distinct constructs rather than redundancy [4, 28]. Domains reflecting caregiving burden and psychosocial strain showed stronger associations with generic quality of life, whereas healthcare system trust demonstrated weaker but meaningful relationships. This pattern is consistent with the conceptualisation of healthcare system trust as a system-level determinant of parental quality of life, distinct from individual psychological well-being. The inclusion of this dimension enhances the ecological validity of the QOLA by acknowledging that parental experiences are embedded within institutional and structural contexts [24].

Cross-national analyses indicated that the overall measurement structure and patterns of association were broadly comparable across countries, supporting the relevance of the assessed constructs in different European contexts. Although formal measurement invariance testing was not feasible due to sample size constraints, the absence of substantial or inconsistent country effects suggests that the QOLA captures core dimensions of parental quality of life that are not strongly culture specific. However, formal measurement invariance testing in larger samples will be important before making precise cross-country comparisons, to establish that observed differences reflect parental quality of life rather than differences in how the QOLA functions across cultures, as demonstrated in recent cross-cultural validation work [16]. The present findings nevertheless, according to COSMIN guidance, are informative for preliminary cross-cultural applicability, particularly in rare disease research where large samples are difficult to obtain [21, 30].

### Limitations

Several limitations should be considered. Although the sample size was appropriate for psychometric validation in a rare disease context, it did not allow formal testing of measurement invariance across countries or subgroups; larger samples are needed to evaluate cross-cultural validity in line with COSMIN recommendations. Recruitment via patient organisations may have resulted in a sample with relatively high engagement or access to care, potentially limiting generalisability to less connected families. As a parent-reported outcome measure, the QOLA reflects caregivers’ subjective perceptions, which may be influenced by individual coping styles or contextual factors.

Responsiveness and minimal important change could not be assessed because there was no intervention or known change between measurement occasions; establishing these properties in future longitudinal and interventional studies will be important for determining the QOLA’s ability to detect meaningful change over time [7, 29]. Finally, the EFA and subsequent CFA were conducted in the same sample, which may limit the generalisability of the factor structure; independent-sample validation is therefore warranted in future studies.

## Conclusion

This study supports the Quality of Life of Parents of Children with Achondroplasia (QOLA) as a valid and reliable instrument for assessing parental quality of life in achondroplasia. By capturing daily caregiving constraints, future- and social-related psychosocial strain, and healthcare system trust, the QOLA addresses dimensions of parental burden that are directly relevant to clinical care and service organisation in rare disease contexts. The use of a condition-specific measure enables more comprehensive assessment of parental needs than generic instruments and may support structured evaluation of family well-being, care coordination, and service delivery. Further research should examine responsiveness and interpretability to support routine clinical and evaluative use.

## Supplementary Information

Below is the link to the electronic supplementary material.

Supplementary Material 1

## Data Availability

The datasets generated and/or analysed during the current study are available from the corresponding author upon reasonable request.
