# Supplementary material for: The QOLA study: psychometric validation of the Quality of Life of Parents of Children with Achondroplasia (QOLA) questionnaire - a multinational study in Germany, Italy, and Portugal
Source: J Patient Rep Outcomes. 2026 Sep 25;10:170. doi: 10.1186/s41687-026-01207-w (PMC13615216; doi:10.1186/s41687-026-01207-w)
Supplement: Supplementary file 1 [file 41687_2026_1207_MOESM1_ESM.docx]

***Supplementary File***

The QOLA Study

**Psychometric Validation of the Quality of Life of Parents of Children with Achondroplasia (QOLA) Questionnaire: A Multinational Study in Germany, Italy, and Portugal**

***Supplementary Table S1. Item-level descriptive statistics of the EUROHIS-QOL-8 at baseline (T1) and retest (T2)***

| **EUROHIS-QOL-8 Item** | **T1 Mean (SD)** | **T1 Min–Max** | **T2 Mean (SD)** | **T2 Min–Max** |
| --- | --- | --- | --- | --- |
| Overall quality of life | 4.03 (0.66) | 2.00–5.00 | 3.94 (0.63) | 2.00–5.00 |
| Satisfaction with health | 3.62 (0.78) | 2.00–5.00 | 3.55 (0.73) | 2.00–5.00 |
| Energy for everyday life | 3.40 (0.85) | 1.00–5.00 | 3.36 (0.79) | 2.00–5.00 |
| Satisfaction with daily activities | 3.58 (0.81) | 2.00–5.00 | 3.49 (0.75) | 2.00–5.00 |
| Satisfaction with self | 3.77 (0.74) | 2.00–5.00 | 3.69 (0.70) | 2.00–5.00 |
| Satisfaction with personal relationships | 3.89 (0.70) | 2.00–5.00 | 3.80 (0.66) | 2.00–5.00 |
| Satisfaction with financial situation | 3.41 (0.86) | 1.00–5.00 | 3.35 (0.80) | 1.00–5.00 |
| Satisfaction with living conditions | 3.86 (0.72) | 2.00–5.00 | 3.78 (0.69) | 2.00–5.00 |

**Note.** Items are rated on a five-point Likert scale, with higher scores indicating better perceived generic quality of life. T1 sample size = 149; T2 sample size = 77. Minor differences in sample size across items reflect occasional item-level missingness. The global quality-of-life item was optional, resulting in higher non-response at both time points.

***Supplementary Table S2. Convergent validity of QOLA domain scores with EUROHIS-QOL-8 at baseline (T1) and retest (T2)***

|  | **Pooled Sample** | | | | **Germany** | | | | **Italy** | | | | **Portugal** | | | |
| --- | --- | --- | --- | --- | --- | --- | --- | --- | --- | --- | --- | --- | --- | --- | --- | --- |
|  | **T1 (N= 149)** | | **T2 (N = 77)** | | **T1 (N= 55)** | | **T2 (N = 14)** | | **T1 (N= 43)** | | **T2 (N = 25)** | | **T1 (N= 50)** | | **T2 (N = 38)** | |
| **QOLA Domain** | ***r*** | ***p*** | ***r*** | ***p*** | ***r*** | ***p*** | ***r*** | ***p*** | ***r*** | ***p*** | ***r*** | ***p*** | ***r*** | ***p*** | ***r*** | ***p*** |
| Healthcare System Trust | −0.246 | .031 | −0.150 | .194 | −0.408 | .002 | −0.084 | .775 | −0.196 | .209 | −0.197 | .344 | −0.403 | .004 | −0.308 | .060 |
| Care System Strain | −0.354 | .002 | −0.414 | < .001 | −0.470 | < .001 | −0.547 | .043 | −0.338 | .027 | −0.601 | .001 | −0.504 | < .001 | −0.415 | .009 |
| Physical Strain of Caregiving | −0.595 | < .001 | −0.691 | < .001 | −0.679 | < .001 | −0.631 | .015 | −0.526 | < .001 | −0.718 | < .001 | −0.671 | < .001 | −0.758 | < .001 |
| Mental Health and Emotional Burden | −0.413 | < .001 | −0.547 | < .001 | −0.321 | .017 | −0.592 | .026 | −0.354 | .020 | −0.634 | < .001 | −0.476 | < .001 | −0.435 | .006 |
| Social Integration and Participation | −0.411 | < .001 | −0.359 | .001 | −0.563 | < .001 | −0.433 | .122 | −0.337 | .027 | −0.570 | .003 | −0.484 | < .001 | −0.349 | .032 |
| Coping | −0.252 | .027 | −0.377 | < .001 | −0.626 | < .001 | −0.587 | .027 | −0.377 | .013 | −0.435 | .030 | −0.270 | .058 | −0.409 | .011 |
| Family Dynamics and Support | −0.292 | .010 | −0.357 | .001 | −0.433 | < .001 | −0.644 | .013 | −0.289 | .060 | −0.526 | .007 | −0.467 | < .001 | −0.553 | < .001 |
| Worries about the Future and Social Relationships | −0.249 | .029 | −0.263 | .021 | −0.155 | .260 | −0.428 | .127 | −0.174 | .265 | .002 | .993 | −0.094 | .515 | −0.156 | .348 |

**Note.** Pearson correlation coefficients (*r*) are shown. Higher QOLA domain scores reflect greater burden or impairment, whereas higher EUROHIS-QOL-8 scores indicate better quality of life; therefore, negative correlations were expected. *p-values* are two-tailed.
